# High sensitivity C reactive protein, fibrinogen levels and the onset of major depressive disorder in post-acute coronary syndrome

**DOI:** 10.1186/s12872-015-0015-3

**Published:** 2015-03-18

**Authors:** Marianne Lafitte, Sandrine Tastet, Paul Perez, Marie-Aimée Serisé, Anne-Sophie Grandoulier, Bruno Aouizerate, Igor Sibon, Lucile Capuron, Thierry Couffinhal

**Affiliations:** CHU de Bordeaux, Centre d’Exploration, de Prévention et de Traitement de l’Athéroclérose, CEPTA, Hôpital Cardiologique du Haut-Lévêque, Avenue de Magellan, 33604 PESSAC Cedex, F-33000 Bordeaux, France; CHU de Bordeaux, Unité de Soutien Méthodologique à la Recherche Clinique et Epidémiologique, F-33000 Bordeaux, France; CHU de Bordeaux, Pôle Universitaire de Psychiatrie, F-33000 Bordeaux, France; CHU de Bordeaux, Unité Neurovasculaire, F-33000 Bordeaux, France; INRA, Nutrition et Neurobiologie intégrée, UMR 1286, F-33000 Bordeaux, France; Univ. Bordeaux, Adaptation cardiovasculaire à l’ischémie, U1034, F-33600 Pessac, France; INSERM, Adaptation cardiovasculaire à l’ischémie, U1034, F-33600 Pessac, France

**Keywords:** Depression, Inflammatory marker, CRP, Fibrinogen, Acute coronary syndrome, Atherosclerosis

## Abstract

**Background:**

Major depression disorder (MDD) is a common condition in patients suffering from acute coronary syndrome (ACS), and depression is a risk factor for mortality following an ACS. Growing evidence suggests that there is an intricate interplay between atherosclerosis, inflammation and depression. The aim of this study was to investigate the role of atherosclerosis-induced inflammation in the mediation of MDD.

**Methods:**

87 patients without depression were recruited at the time of an ACS, evaluated at 3 and 7 days and followed at 1, 3 and 9 months for the occurrence of a MDD as assessed by structured interviews (MINI). At each time point, they were monitored for inflammatory markers (high sensitivity C Reactive Protein {hsCRP} and fibrinogen), cardiovascular risk factors and atherosclerosis burden. Association between possible predictive characteristics and depression was assessed using a multivariable logistic regression model.

**Results:**

The overall incidence of MDD, in this population, was 28.7% [95% CI: 19.5 – 39.4] during the 9-month follow up period. Elevated hsCRP was not associated with depression onset after an ACS (adjusted OR: 1.07 [0.77 - 1.48]; p = 0.70), and similarly no association was found with fibrinogen. Furthermore, we found no association between hsCRP, fibrinogen or atherosclerosis burden at any time-point, and the occurrence of a MDD (or HDRS-17 and MADRS). The only factor associated with depression occurrence after an ACS was a previous personal history of depression (adjusted OR: 11.02 [2.74 to 44.34]; p = 0.0007).

**Conclusions:**

The present study shows that after an ACS, patients treated with optimal medications could have a MDD independent of elevated hsCRP or fibrinogen levels. Personal history of depression may be a good marker to select patients who should be screened for depression after an ACS.

**Electronic supplementary material:**

The online version of this article (doi:10.1186/s12872-015-0015-3) contains supplementary material, which is available to authorized users.

## Background

Major depression disorder (MDD) is a common condition in patients suffering from acute coronary syndrome (ACS), affecting approximately 20% of patients during hospitalization and a similar proportion within the first year after ACS [1,2]. Depressive symptoms, even in the absence of formal diagnosis of MDD, are strong independent predictors of cardiovascular morbidity and mortality after ACS [3,4].

Understanding the mechanisms underlying the onset of depression, and identifying early markers that predict its occurrence in patients after ACS, could have major clinical implications in both the optimal management of depression and secondary prevention of coronary artery disease (CAD) [5].

However, the mechanisms specifically involved in the association between cardiovascular disease and depression have not been clearly established. Growing evidence suggests that there is an intricate interplay between atherosclerosis, inflammation and depression [6]. These inter-relations have been reported in the literature in different ways: depression is frequently diagnosed in patients with CAD and MDD is a powerful risk factor for CAD events [7-9]. Atherosclerosis is fundamentally an inflammatory disease and inflammatory markers are powerful predictors of CAD events [10,11]. MDD is associated with an increased level of markers of inflammation and can be induced by pro-inflammatory treatment or cytokine therapy in medically ill patients [12-18].

Therefore, it would be reasonable to hypothesize that the link between CAD and depression might be mediated by inflammation. However, the causal and temporal mechanisms underlying the inter-relationships between CAD, inflammation and depression have not been well characterized.

The primary objective of the present study was to investigate the prognostic value of hsCRP or fibrinogen (as surrogate markers of inflammation) in detecting new MDD, after an ACS. For this purpose, we excluded patients who had a diagnosis of depression at study entry, or were receiving treatment for depression. We hypothesized that high, followed by low-grade systemic inflammation, after an ACS (as measured by serum hsCRP and fibrinogen levels), could induce and be a biological marker able to predict depression. The secondary objective was to investigate other factors that might predict the development of depression after an ACS.

## Methods

### Patients

Between May 2006 and September 2007, a total of 146 potentially eligible patients were admitted to our department 2–3 days after an acute coronary syndrome (ACS). The present study enrolled patients 30–75 year old with an ACS, defined as previously reported [19]. Specific exclusion criteria with respect to depression, anti-inflammatory drugs and inflammatory diseases, were as follows:

- ▪A previous history of either major depressive disorder (MDD) within the last 6 months or treatment for depression within the last 6 months; a current diagnosis of MDD and/or ongoing treatment for depression at the time of hospitalization for ACS. Patients who had a major psychiatric disorder other than affective disorders were also excluded (e.g., schizophrenia, dementia, present psychotic episode).
- ▪A treatment by steroid, COX-2 selective inhibitor or other non-steroidal anti-inflammatory drug (aspirin > 325 mg) for more than 7 days before hospitalization for ACS.
- ▪A surgery in the last month, presence of a severe systemic or infectious disease, autoimmune disorder, inflammatory disorder, or HIV, treatment with dialysis, or a malignancy with decreased life expectancy.

Other exclusion criteria included unstable medical or neurologic condition and patients who were unable to communicate reliably (e.g., because of cognitive dysfunction or not speaking French).

The Ethics Committee review board of the hospital (CPP SOOM III, Bordeaux) approved the study protocol, and all participants provided informed consent prior to participation. All standard of care cardiovascular treatments were permitted during the trial. Concomitant medication, including steroids, COX-2 selective inhibitors and other non-steroidal anti-inflammatory treatments were recorded during the study.

### Acute phase management

ACS was defined and treated according to established guidelines [20]. A coronary angiogram was performed on each patient included in the study, allowing precise evaluation of coronary lesions, and optimized acute phase management. The most suitable treatment was delivered during the first week, in compliance with current guidelines [20].

### Psychiatric assessment

- ▪The Mini-international neuropsychiatric interview (MINI) was used to assess the incidence of MDD. MINI is a short structured clinical interview, which enables researchers to make a diagnosis of psychiatric disorders according to DSM-IV or ICD-10 [21]. Patients were classified as having MDD or not based upon results of the MINI.
- ▪The Hamilton Depression Rating Scale (HDRS-17), is a clinician-administered questionnaire assessing the severity of depressive symptoms, including low mood, insomnia, agitation, anxiety and weight loss [22]. HDRS-17 assesses severity of, and change in, depressive symptoms over time.
- ▪The Montgomery-Åsberg Depression Rating Scale (MADRS), is a ten-item clinician-administered questionnaire assessing the severity and evolution of depressive symptoms [22]. The MADRS is one of the most frequently used and validated observer-rated depression scales.

### Procedure

Patients meeting the inclusion criteria were invited to participate as soon as they were medically stable and had been informed of their diagnosis, on average 2 days after their ACS (range, 2–4 days). After explaining the study and obtaining written informed consent, a research psychologist (S.T.) conducted all baseline psychiatric interviews, and gathered routine demographic data, including age, gender, education, living arrangements, current partner status, socioeconomic status and employment status (employed or unemployed). Information on history of familial and personal depression, or other psychiatric illness was collected during hospitalization.

During the assessment period, if the MINI was positive for a diagnosis of MDD, the patient was excluded from the study. If it was negative, the other psychiatric interviews were performed, as well as an evaluation of hsCRP and fibrinogen levels along with other classical risk factors.

All patients benefited from the CEPTA program with an optimization of secondary prevention measures and educational classes as previously described [19,23].

Patients were followed at 1, 3 and 9 months after ACS. At each time point, cardiovascular risk factors, treatment, levels of hsCRP and fibrinogen were recorded, and psychiatric interviews were performed. The cardiologists and the care team were blinded to the depression status over the 9 month follow-up period. Patients with depressive symptoms were not treated with antidepressant medication, due to the lack of evidence of a clear benefit of depression screening, and of antidepressant medication in this patient population, on cardiovascular disease (CVD) outcome. Instead these patients were sent to a mental health professional for psychotherapy alone.

### End point

To assess the incidence of MDD, the psychologist administered the MINI at 3 days and 1, 3 and 9 months, after an ACS. The psychologist also conducted the HDRS-17 and MADRS interviews at 3 days, 1, 3 and 9 months, to score the intensity and evolution of depression symptoms.

To assess the inflammatory status, hsCRP and fibrinogen levels were measured at 3 and 7 days, 1, 3 and 9 months. hsCRP was measured by immuno-turbidimetry, with a detection limit of 0.02 mg/L (Rock Diagnoses). The assays were performed blinded to patients’ depression status; plasma samples drawn at baseline were frozen at −70°C for subsequent measurement of inflammatory [19] markers.

The 9-month survival status was determined for all patients after their ACS. Cause of death was established from hospital and general practitioner records and death certificates. Deaths were classified as cardiac or non-cardiac by a consulting cardiologist blinded to baseline data.

### Statistics

Continuous variables were described by mean and standard deviation, and compared using Student’s t tests. Categorical variables were described by numbers and proportions, and compared using Chi-square tests. Comparisons were considered statistically significant when the p value was <0.05, and corrected according to the Bonferroni rule in case of multiple testing. The incidence rate of MDD was estimated by the proportion of patients presenting with at least one positive MINI at any time point divided by the total number of patients followed during the 9-month period after an ACS. Ninety-five percent confidence intervals (95% CI) of proportions and incidence rates were calculated by the exact binomial method. The association between patient characteristics and the diagnosis of MDD was assessed by logistic regression models, using standard recommended methods. The strength of the association between predictors and MDM was estimated by calculating the odds ratios (OR) and their 95% CIs. Linearity of the logit was systematically checked for every continuous variable. A limited number of possible predictive variables were chosen on the basis of previous reports (age, female gender, familial and personal history of depression, low socio-economic level, living alone, atherosclerosis burden, LDL cholesterol, metabolic syndrome, smoking and γGT). Their association with the occurrence of depression was first tested in univariate logistic regression analysis. Then, all variables associated with depression with a p value < 0.20 were introduced into the multivariable model. The variable of interest, hsCRP level at discharge, was forced into the model. The full model was kept without further selection, as the aim was to obtain an estimate of the association of hsCRP with depression, adjusted based on possible confounding factors.

In cases where there was missing data for either hsCRP or MINI, sensitivity analyses were carried out. Exploratory analysis of the association between the evolution of depressive symptoms, as assessed by MADRS, and the evolution of hsCRP was carried out using a mixed linear model.

All analyses were carried out using SAS® software v9.1.3 (SAS Institute Inc., Cary, North Carolina, USA).

## Results

### Incidence and time course of depressive symptoms

**Figure 1:**
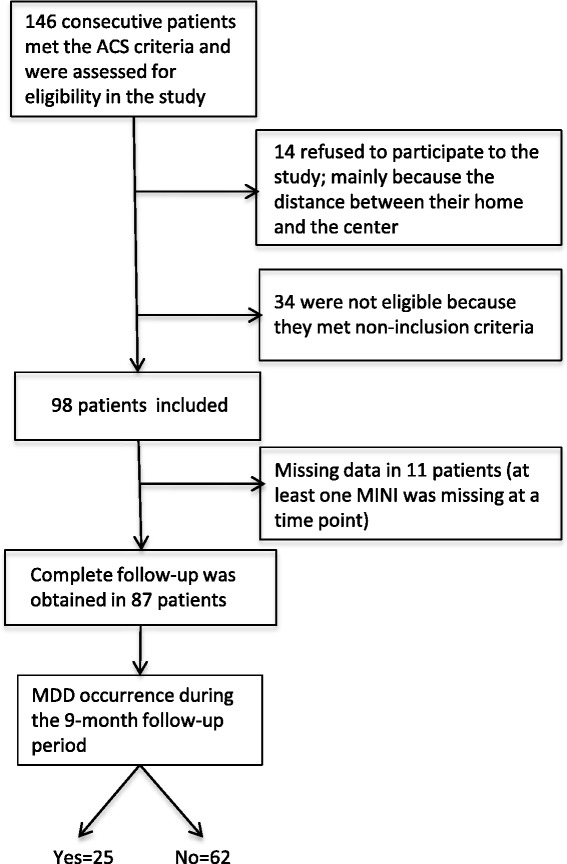
**Flow chart of the study population.**

The study population flow chart is shown in Figure 1. At the time of ACS, none of the 87 patients included in the study had a MDD, or a history of MDD less than 6 months before their initial hospitalization. New onset of depression was recorded in 8.0% of the patients at 1 month, 10.3% at 3 months and 10.3% at 9 months. A patient was categorized as part of the “depression group” if he or she had a MDD at any time point during follow up, as assessed by the MINI. The overall 9-month incidence of a MDD was 28.7% [95% CI: 19.5 – 39.4]. At 9 months, 19.5% of patients (prevalence) still met the criteria for diagnosis of depression.

### Patients’ characteristics according to depressive symptoms status

There were no significant differences in general characteristics and the risk factor profile, between patients who remained free of MDD (non-depression group) versus those who developed MDD (depression group) at any of the follow up time points, with the exception of prior history of depression (Table 1 and Additional file 1: Table S1). There were significantly more patients in the depression group who reported having a prior history of depression, than in the non-depression group (88%, vs 37.1% p < 0.0001).

**Table 1:** **General characteristics, atherosclerosis burden, acute phase management and medical treatments at discharge, grouped according to the depression status over a 9-month follow-up after ACS**

	**No Depression 71.3%**	**Depression 28.7%**
**General characteristics**		
Mean Age ± sd (years)	49.0 ± 8.8	52.2 ± 9.2
Female gender (%)	6.4	16.0
Personal history of CV disease (%)	8.1	16.0
Family history of CV disease (%)	17.7	28.0
Personal history of depression (%)	37.1	88.0 **
Familial history of depression (%)	25.8	20.0
Live alone (%)	17.7	24.0
Widow(er)/divorcee/separated (%)	45.1	48.0
Low Socio-economic level (%)	8.0	16.0
**Atherosclerosis Burden**		
Mean number of coronary vessels >50% stenosis	1.4+/−0.8	1.6+/−0.6
ABI < 0.9 (%)	11	7
Carotid stenosis >20% (%)	18.6	22.2
IMT > 0.7 mm (%)	63	60
**ACS treatment**		
Revascularisation (%)	91.9	92.0
Conventional Treatment (%)	8.1	8.0
**Post ACS medications at discharge**		
Beta-blocker and/or calcium antagonist	96.7%	96.0%
Cholesterol-lowering medication	93.5%	100.0%
Antiplatelet therapy or warfarin	100.0%	100.0%
ACE inhibitor or Angiotensin II receptor agonist	100.0%	96.0%
Combination of anti-ischemic/antiplatelet/Lipid-lowering drug	90.3%	96.0%

*p values of tests comparing the “No depression” and “Depression” groups (Student’s t test for age, Chi square tests for proportions). * p < 0.05; ** p < 0.01.

There was no association found between depressive symptom status and atherosclerosis burden (Table 1), or major cardiovascular events (such as reinfarction, stroke or death) during the 9 months follow-up period (3 ACS and 1 stroke occurred in the non-depression group vs. no event in the depression group).

Acute phase management and optimal medical treatment were similar in both groups of patients (MDD vs. no depression) at discharge (Table 1) and during the follow-up period (not shown).

### Association between hsCRP and occurrence of major depressive disorder

**Figure 2:**
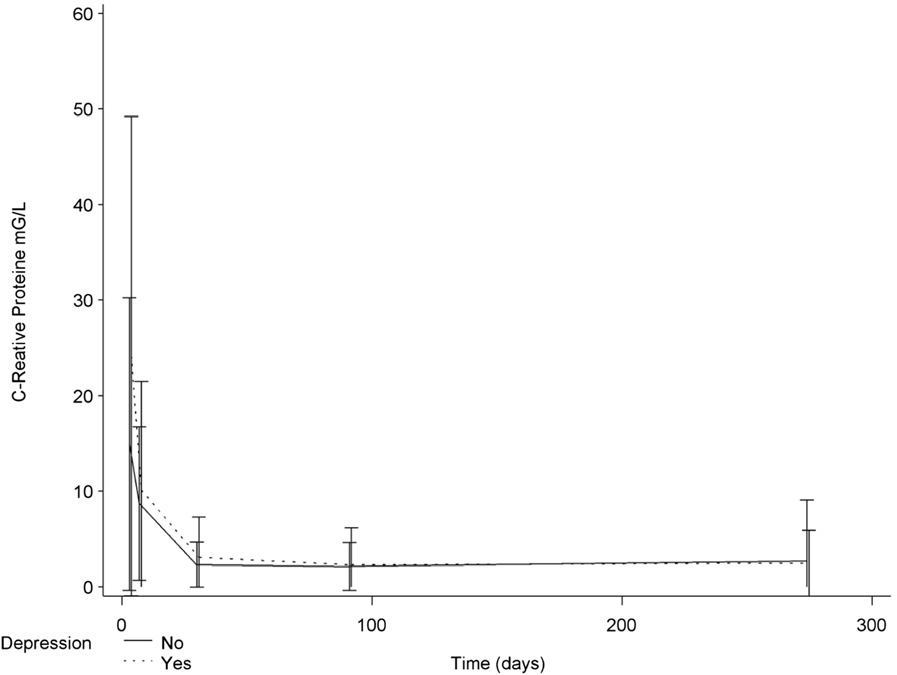
**Comparison of hsCRP evolution (mean+/− SD) in the group of patient with no depression versus patients with depression.**

According to HDRS-17 and MADRS scales, the majority of these MDD patients were classified as having mild to moderate depression (Table 2). No difference in hsCRP levels was detected between the depression and non-depression groups at any time-point (Table 2). The evolution of mean hsCRP levels was similar in the two groups and even individual values did not show any trend (Figure 2 and Additional file 1: Figures S1 and S2). In univariate, unadjusted analysis, hsCRP measured at day 7 was not associated with the occurrence of a MDD during the 9-month follow-up period after the ACS. The odds ratio for a MDD during the first 3 months was 0.96 (95% CI: 0.88 - 1.05; p = 0.37) and the odds ratio for a MDD during the first 9 months was 1.02 (95% CI: 0.97 - 1.07; p = 0.53) (Table 3). We also estimated the association between hsCRP measured at 1 and 3 months and the occurrence of a MDD during the first 3 or 9 months, but all odds ratios were close to 1 and not statistically significant (Table 3). In multivariable analysis, i.e. when taking into account other characteristics, hsCRP was not associated with the occurrence of MDD during the first nine months period after ACS. The adjusted OR was 1.07 (95% CI: 0.77 -1.48; p = 0.70) (Table 4).

**Table 2:** **Depression severity and intensity, as measured with HDRS-17 and MADRS scales (mean ± sd) and evolution of the inflammatory profile, as measured by hsCRP and fibrinogen levels, according to the depression status over a 9-month follow-up period after an ACS**

	**D7**		**M1**		**M3**		**M9**	
	**No Depression 71.3%**	**Depression 28.7%**	**No Depression 71.3%**	**Depression 28.7%**	**No Depression 71.3%**	**Depression 28.7%**	**No Depression 71.3%**	**Depression 28.7%**
MADRS	5.5 ± 3	6.9 ± 4	5.7 ± 4	13.9 ± 8*	6.2 ± 4	18.2 ± 8**	5.5 ± 5	21.0 ± 8**
HDRS-17	6.7 ± 3	9.2 ± 4	9.0 ± 5	14.0 ± 7*	8.6 ± 5	17.2 ± 7*	7.5 ± 5	20.1 ± 5**
hs-CRP (mg/l)	8.7 ± 8.0	10.1 ± 11.0	2.30 ± 2.3	3.05 ± 4.2	2.12 ± 2.5	2.27 ± 3.8	2.65 ± 6.4	2.46 ± 3.4
Fibrinogen (g/l)	5.22 ± 1.1	5.62 ± 1.2*	3.97 ± 0.7	4.18 ± 1.1	3.65 ± 0.6	3.62 ± 1.0	3.69 ± 0.8	4.16 ± 0.8*

*p values of tests comparing the “No depression” and “Depression” groups (Student’s t test for age, Chi square tests for proportions). *p < 0.05; **p < 0.01.

**Table 3:** **Univariate association between hsCRP or fibrinogen measured at 3 or 7 days, 1 and 3 months (as indicated) and the occurrence of a MDD during the first 3 or 9 months**

**Variable**	**Time of depression**	**OR**	**IC 95%**	**p-value**
hsCRP D7	In the first 3 months	0.96	[0.88 ; 1.05]	0.37
hsCRP D7	In the first 9 months	1.02	[0.97 ; 1.07]	0.53
hsCRP M1	In the first 3 months	0.90	[0.67 ; 1.20]	0.46
hsCRP M1	In the first 9 months	1.08	[0.93 ; 1.26]	0.32
hsCRP M3	In the first 3 months	0.85	[0.61 ; 1.20]	0.36
hsCRP M3	In the first 9 months	1.02	[0.86 ; 1.20]	0.84
**Variable**	**Time of depression**	**OR**	**IC 95%**	**p-value**
Fibrinogen D3	In the first 3 months	1.01	[0.60 ; 1.69]	0.97
Fibrinogen D3	In the first 9 months	1.38	[0.92 ; 2.08]	0.12
Fibrinogen M1	In the first 3 months	0.78	[0.39 ; 1.56]	0.49
Fibrinogen M1	In the first 9 months	1.22	[0.72 ; 2.09]	0.46
Fibrinogen M3	In the first 3 months	0.70	[0.33 ; 1.51]	0.37
Fibrinogen M3	In the first 9 months	0.89	[0.48 ; 1.66]	0.71

**Table 4:** **Multivariable analysis of association between the depression status over a 9-month follow-up period after ACS and variables recorded at day 7**

**Variables measured at D7**	**OR [95% CI]**
***hsCRP at D7***	1.07 [0.77 - 1.48]
***Gender (women)***	1.55 [0.21 – 11.35]
***Personal history of depression***	11.02 [2.74 - 44.34]*
***γGT > = 50***	2.30 [0.67 – 7.89]
***HDRS-17***	1.08 [0.92 - 1.26]

*p < 0.001.

Statistics were done on 87 of 98 patients originally included. Eleven patients were not analysed because at least one MINI was missing at a time point. Sensitivity analyses were carried out by replacing missing values on MINI and hsCRP according to different strategies. All 11 missing depression statuses were replaced by absence, then by presence of depression, without generating a significant association between hsCRP D7 and 9-month depression occurrence. When the 9 missing values of hsCRP D7 were replaced by the highest hsCRP value (46.3) for the patients of the depressed group and the lowest hsCRP value (0.3) for the patients of the non-depressed group, no significant association was found.

Moreover, we found no association between hsCRP at day 7 and either HDRS-17 or MADRS scores at months 1, 3 and 9 (not shown). We also used a mixed linear model for studying the association between the evolution of depressive symptoms, as assessed by MADRS, and the evolution of hsCRP, between inclusion and one-month follow-up. No association was found (p = 0.69). The evolution of hsCRP values was quite similar after the one-month follow-up (Figure 2).

Patients diagnosed as depressed at any time point, had higher fibrinogen levels at baseline and at their 9 months assessment, than their non-depressed counterparts, but this difference was not statistically significant at 1 and 3 month assessment (Table 2). Moreover, there was no significant association between fibrinogen values and the occurrence of depression during different periods of time, as analyzed by univariate logistic regression (Table 3).

### Predictive factors of a major depressive disorder during the 9 months following an ACS

We evaluated the association between quantitative and non-quantitative characteristics and CV risk factors of the population, as well as parameters of atherosclerosis burden, with MDD occurrence. A limited number of possible predictive variables were chosen on the basis of previous reports in the literature. In univariate (Additional file 1: Table S2) as well as in the above-mentioned multivariable analyses (Table 4), only the personal history of depression (more than 6 month before ACS) was significantly associated with the onset of a depressive episode during the 9-month follow-up period, with an OR of 11.02 (95% confidence interval [CI], 2.74 to 44.34; p = 0.0009) adjusted for gender, hsCRP, Gamma-GT at day 7 and HDRS-17 score at inclusion (Table 4).

## Discussion

The aim of this study was to determine whether or not atherosclerosis-induced inflammation plays a role in the mediation of MDD and, to examine predictive markers of depression after an ACS. To our knowledge, this is the first study to assess the prognostic importance of hsCRP (and fibrinogen) in the development of MDD after an ACS. This study included only patients without a current, or recent (within 6-months) past history, of depression, as determined by a structured clinical interview. All patients were provided with optimal treatment for their cardiovascular disease.

Although the incidence of depression was high, we found no association between hsCRP or fibrinogen and MDD after an ACS. Therefore, the present data do not support the hypothesis that hsCRP or fibrinogen (as surrogates of inflammation) are an indispensable trigger of depression after ACS in a population treated with optimal medication nor that the reported prognostic impact of depression can be explained by these markers. The only factor associated with depression occurrence after an ACS was a previous personal history of depression.

### Time course and incidence of depression

A comprehensive literature review of hospitalized post-MI patients, revealed a prevalence rate for MDD of approximately 20%, when measured using clinical interviews [24,25]. Our results show that, in the 9 months following an ACS, 28.7% of patients developed MDD. We chose to exclude patients with a history of depression within the last six months and those who were depressed at the time of initial hospitalization for their ACS [26]. Therefore, this study highlights the high incidence of new cases of MDD after an ACS and the long delay in occurrence after the onset of ACS.

### Inflammation as assessed by levels of hsCRP and fibrinogen is not likely to be the mechanism underlying depression

Depression can be a risk factor for the development of CAD, and often exacerbates the outcome, when present in patients with established CAD [7-9]. Atherosclerosis is fundamentally an inflammatory disease and inflammatory markers are powerful predictors of CAD events [10,11]. Depression and inflammation have each been independently associated with CAD [6,12,13,15,17,18,27-33]. The interplay between depression, inflammation, and CVD or mortality is complex, and may be analyzed in several different ways based on literature [6-13,15,17,18,27-32] : (i) depression may induce inflammation, and the latter mediate independently the relationship between depression and CVD; (ii) both depression and inflammation may cause CVD but through separate pathways, being synergistic or not; (iii) depression and inflammation may have a common precursor that is linked to CVD; or (iv) inflammation may cause depression and each may or may not have a causal role on CVD [6].

The primary focus of this study was to determine whether or not inflammation may cause depression, because of the importance of inflammation in atherosclerosis. In this study, we found no significant association between atherosclerosis burden, hsCRP or fibrinogen levels at any time point and depressive symptoms after an ACS. The point estimation of the OR was close to one (1.07), favoring the conclusion of an absence of association. However, due to the limited number of patients, the precision of the estimate was low (95% confidence interval: 0.77 - 1.48). Various sensitivity analyses were conducted by imputing extreme values for missing data but none resulted in an association between hsCRP and MDD. Exploratory analyses did not show an association between the evolution of hsCRP and the evolution of depression scales. These arguments reinforce the confidence in this null result. Therefore, this study supports the hypothesis that patients after an ACS may have a MDD independent of an elevated hsCRP or fibrinogen level.

These data are in contrast to epidemiological and clinical studies which have suggested that inflammation precedes the occurrence of depression as in patients with cancer treated with immunotherapy [17,18,27,29,33,34]. Previous studies on atherosclerosis, inflammation and depression [6] have been limited to selected clinical samples and/or have failed to control for important confounding factors [12]. It is not clear to what extent these associations are due to coexisting cardiovascular risk factors and comorbidity, or the greater severity of atherosclerotic disease among patients with depression [6]. There are several factors which may explain why our results are not consistent with these previous reports of the association between ACS, depression, and biomarkers of inflammation.

In our study, we used a structured diagnostic interview, the gold standard for defining clinical depression, while very few reports in the literature have used an interview-based study to examine the relationships between CVD, depression and inflammation. The majority of studies have used self-reported questionnaires such as the Beck Depression Inventory, to define depression, which are not specific for clinical depression. Thus, an elevated score may reflect transient sadness, anxiety, other psychiatric disorders, general distress secondary to a life-threatening experience, or even the symptoms of the medical illness [35].

In our study, patients who had a diagnosis of depression at study entry were excluded. If individuals with a known diagnosis of depression were not excluded, the cohorts would have had a greater prevalence of depression, than those where only individuals without clinically recognized depression were screened, and the relationship with inflammation could be skewed [26].

We also assessed depression at four time points, where as in the majority of studies examining the interaction of depressive symptoms with inflammation in patients with ACS have measured depressive symptoms only at the time of the hospitalization for ACS. Depressive symptoms at this stage may reflect transient “reactive” depression that may undergo spontaneous remission [5].

Finally, the association between depression and inflammation reported in the literature could be explained by inconsistencies in monitoring risk factors for CVD, co-morbidity, and CAD severity [6]. In our study we were able to control these factors, which could confound the association between depression and inflammation [6]. One difficulty in interpretation of reports in the literature, is the impact of medication such as statins [36]. In addition to their effects on cholesterol synthesis, statins also act as immunomodulators with pronounced anti-inflammatory properties [37]. Because of the optimal medical treatment provided to all patients during the 9 month follow-up period, we were able to minimize these confounding factors.

### Identifying a marker of depression onset after ACS to improve screening

Due to the negative impact of depression on CVD outcome, in patients with CAD, identifying the onset of a MDD is particularly relevant for prevention and intervention [25,26,38-40].

Among all the markers tested in our model only personal history of depression was strongly associated with the occurrence of a MDD during the 9 months following an ACS. Personal history of depression identified patients with a greater vulnerability that were prone to depression. This marker is easy to assess, and may allow physicians to determine which patients could benefit from depression screening.

### Limitation of the study

We acknowledge that this study has a limited number of patients that limit the power of some of statistical analyses and conclusions, namely in showing associations between clinical parameters and MDD occurrence (i.e. interaction between CRP and depression depending on the gender).

It is worth noting that levels of hsCRP and fibrinogen may not recapitulate all inflammatory processes, namely central nervous system inflammation. On the other hand, most often an increase in the levels of TNF-α, IL-1β or IL-6 will result in an increase in hsCRP or fibrinogen levels. However, it may be interesting to introduce other inflammatory markers in future studies.

This study is an exploratory study that may generate new knowledge and hypothesis, but it needs to be replicated.

## Conclusions

The present study demonstrates that after an ACS, patients treated with optimal medication can have a MDD independent of elevated levels of hsCRP or fibrinogen. Our data does not support the hypothesis that inflammation is a trigger for depression after ACS. Personal history of depression may be a good marker to select patients who should be screened for depression after an ACS.

## Additional file

Additional file 1: Table S1. Cardiovascular risk factor profile of the population. Patients were classified according to their depressive disorder status. A patient was considered as “depression” if they had a major depression disorder at any time point during the follow up. (* p<.05, comparison between depression and non depression group for each time point). **Table S2:** Univariate analysis of the association between the depression status over a 9-month follow-up after ACS and variables recorded at day 7. **Figure S1:** hsCRP evolution in patients with no depression, during the 9-month follow-up period. **Figure S2:** hsCRP evolution in patients with at least one MINI positive, during the 9-month follow-up period.

## Abbreviations

ACS: Acute coronary syndrome
CVD: Cardiovascular disease
CAD: Coronary artery disease
hsCRP: High sensitivity C reactive protein
MDD: Major depression disorder
MINI: Mini-international neuropsychiatric interview
HDRS-17: Hamilton depression rating scale
MADRS: Montgomery-åsberg depression rating scale
