# Supplementary material for: High sensitivity C reactive protein, fibrinogen levels and the onset of major depressive disorder in post-acute coronary syndrome
Source: BMC Cardiovasc Disord. 2015 Mar 18;15:23. doi: 10.1186/s12872-015-0015-3 (PMC4436867; doi:10.1186/s12872-015-0015-3)
Supplement: Additional file 1: Table S1. — Cardiovascular risk factor profile of the population. Patients were classified according to their depressive disorder status. A patient was considered as “depression” if they had a major depression disorder at any time point during the follow up. (* p<.05, comparison between depression and non depression group for each time point). Table S2: Univariate analysis of the association between the depression status over a 9-month follow-up after ACS and variables recorded at day 7. Figure S1: hsCRP evolution in patients with no depression, during the 9-month follow-up period. Figure S2: hsCRP evolution in patients with at least one MINI positive, during the 9-month follow-up period. [file 12872_2015_15_MOESM1_ESM.doc]

***Additional file1***

| ***Cardiovascular risk factors*** | **D7** | **D7** | **M3** | **M3** | **M9** | **M9** |
| --- | --- | --- | --- | --- | --- | --- |
|  | **No Depression** | **Depression** | **No Depression** | **Depression** | **No Depression** | **Depression** |
| ***Dyslipidemia*** |  |  |  |  |  |  |
| Total cholesterol (mmol/L) | 4.96+/-1.1 | 5.17+/-1.0 | 3.92+/-0.7 | 4.66+/-1.0 | 4.22+/-0.9 | 4.65+/-1.1 |
| HDL C (mmol/L) | 1.04+/-0.2 | 1.07+/-0.2 | 1.24+/-0.3 | 1.28+/-0.3 | 1.35+/-0.4 | 1.26+/-0.3 |
| LDL C (mmol/L) | 3.16+/-1.0 | 3.2+/-0.8 | 2.09+/-0.6 | 2.54+/-0.8 | 2.31+/-0.7 | 2.55+/-0.9 |
| LDL>1 g/l (%) | 74 | 76 | 20.9 | 48.0 | 32.2 | 36.0 |
| Triglycerides (mmol/L) | 1.71+/-0.7 | 2.05+/-1.3 | 1.30+/-0.5 | 1.88+/-1.0 | 1.25+/-0.4 | 1.93+/-1.1 |
| ***Diabetes*** |  |  |  |  |  |  |
| Fasting glucose (mmol/L) | 5.02+/-0.8 | 5.36+/-0.8 | 5.30+/-0.6 | 5.60+/-0.9 | 5.43+/-0.7 | 5.64+/-0.7 |
| HBA1C (%) | 5.6+/-0.4 | 5.6+/-0.4 | 5.53+/-0.4 | 5.58+/-0.4 | 5.48+/-0.4 | 5.56+/-0.2 |
| Presence of diabetes (%) | 6.4 | 4.0 | 16 | 26 |  |  |
| ***Obesity*** |  |  |  |  |  |  |
| BMI (Kg/m²) | 26.5+/-3.9 | 28.3+/-6.1 | 26.6+/-3.6 | 28.4+/-5.9 | 26.8+/-3.7 | 28.4+/-5.6 |
| Waist circumference (cm) | 95.8+/-11 | 101.0+/-17 | 95.5+/-10.4 | 100.8+/-15.5 | 95.1+/-10.9 | 100.0+/-13.8 |
| Metabolic Syndrome ATP III (%) | 9.6 | 12.0 | 3.2 | 28.0 | 12.9 | 16.0 |
| ***Hypertension*** |  |  |  |  |  |  |
| SBP (mm Hg) | 111+/-12 | 121+/-18 | 111+/-15 | 119+/-16 | 128+/-18 | 130+/-19 |
| DBP (mm Hg) | 64+/-9 | 70+/-13 | 67+/-9 | 71+/-12 | 75+/-11 | 78+/-13 |
| SBP>140 mmHg (%) | 1.6 | 16 | 4.8 | 16.0 | 22.5 | 28.0 |
| ***Current smoker (%)*** | 64.5 | 80 | 21 | 26 |  |  |
| ***Physical activity*** |  |  |  |  |  |  |
| None (%) |  |  | 40 | 63 |  |  |
| Recommended activity (%) |  |  | 60 | 37 |  |  |

**Table S1:** Cardiovascular risk factor profile of the population. Patients were classified according to their depressive disorder status. A patient was considered as “depression” if they had a major depression disorder at any time point during the follow up. (* p<.05, comparison between depression and non depression group for each time point)

| **Qualitative Variables** | **OR** | **IC 95%** | | **p** |
| --- | --- | --- | --- | --- |
| ***Gender*** | 2.76 | 0.63 | 12.04 | **0.17** |
| ***Familial history of depression*** | 0.72 | 0.23 | 2.23 | 0.56 |
| ***Personal history of depression*** | 12.43 | 3.35 | 46.16 | **<0.0001** |
| ***Live alone*** | 1.46 | 0.48 | 4.51 | 0.50 |
| ***Socio-economic level*** | 2.17 | 0.53 | 8.87 | 0.28 |
| ***Three coronary vessels >50% stenosis*** | 0.71 | 0.18 | 2.83 | 0.62 |
| ***Alcohol consumption*** | 1.06 | 0.35 | 3.17 | 0.91 |
| ***GGT ≥ 50*** | 2.16 | 0.84 | 5.54 | **0.11** |

| **Quantitative Variables** | **OR** | **IC 95%** | | **p** |
| --- | --- | --- | --- | --- |
| ***HDRS-17*** | 1.16 | 1.03 | 1.32 | **0.014** |
| ***MADRS*** | 1.11 | 0.97 | 1.27 | **0.114** |
| ***hs-CRP at D7*** | 1.02 | 0.97 | 1.07 | 0.533 |
| ***GGT*** | 1.00 | 1.00 | 1.01 | 0.399 |

**Table S2:** Univariate analysis of the association between the depression status over a 9-month follow-up after ACS and variables recorded at day 7.

**
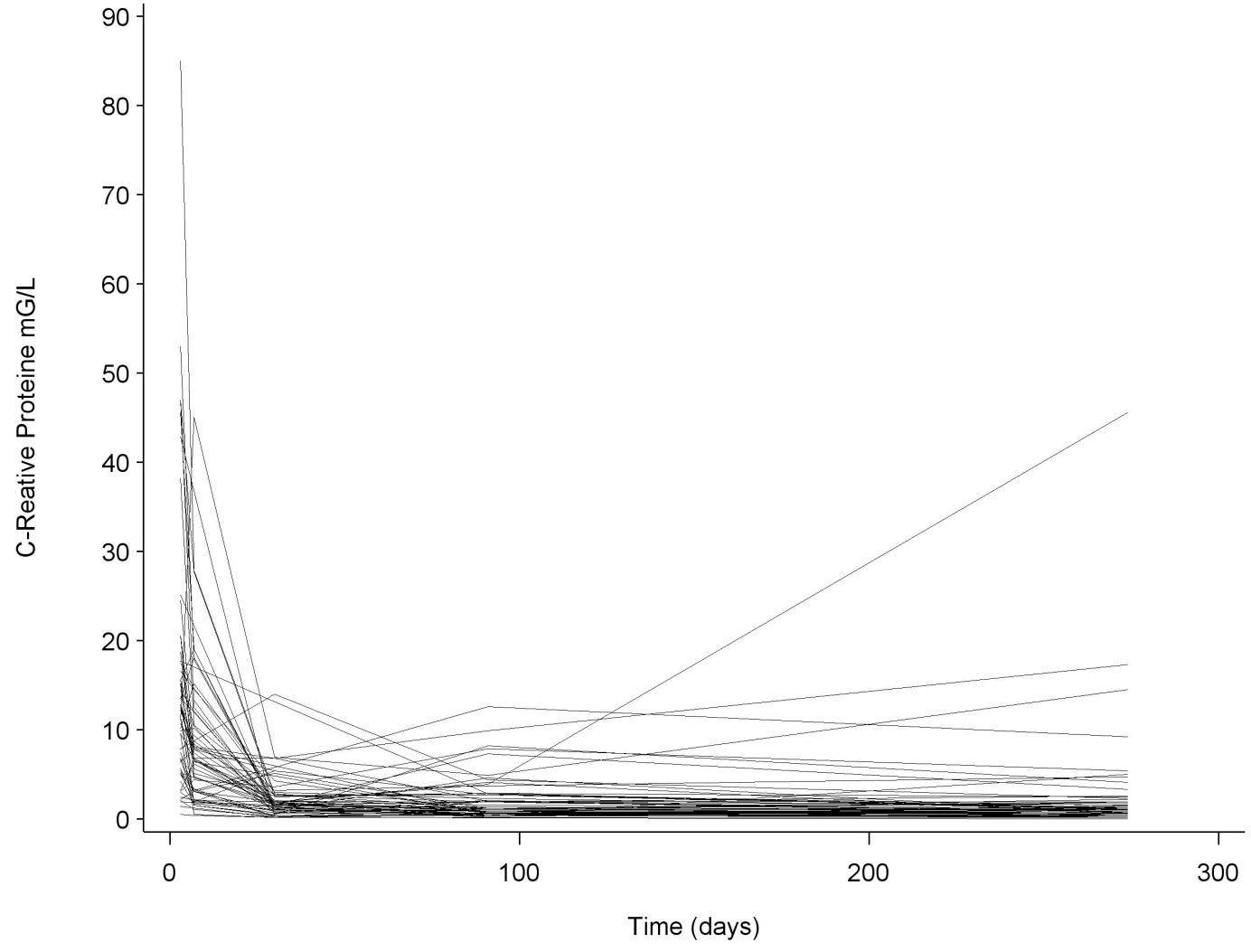
**

**Figure S1**: hsCRP evolution in patients with no depression, during the 9-month follow-up period


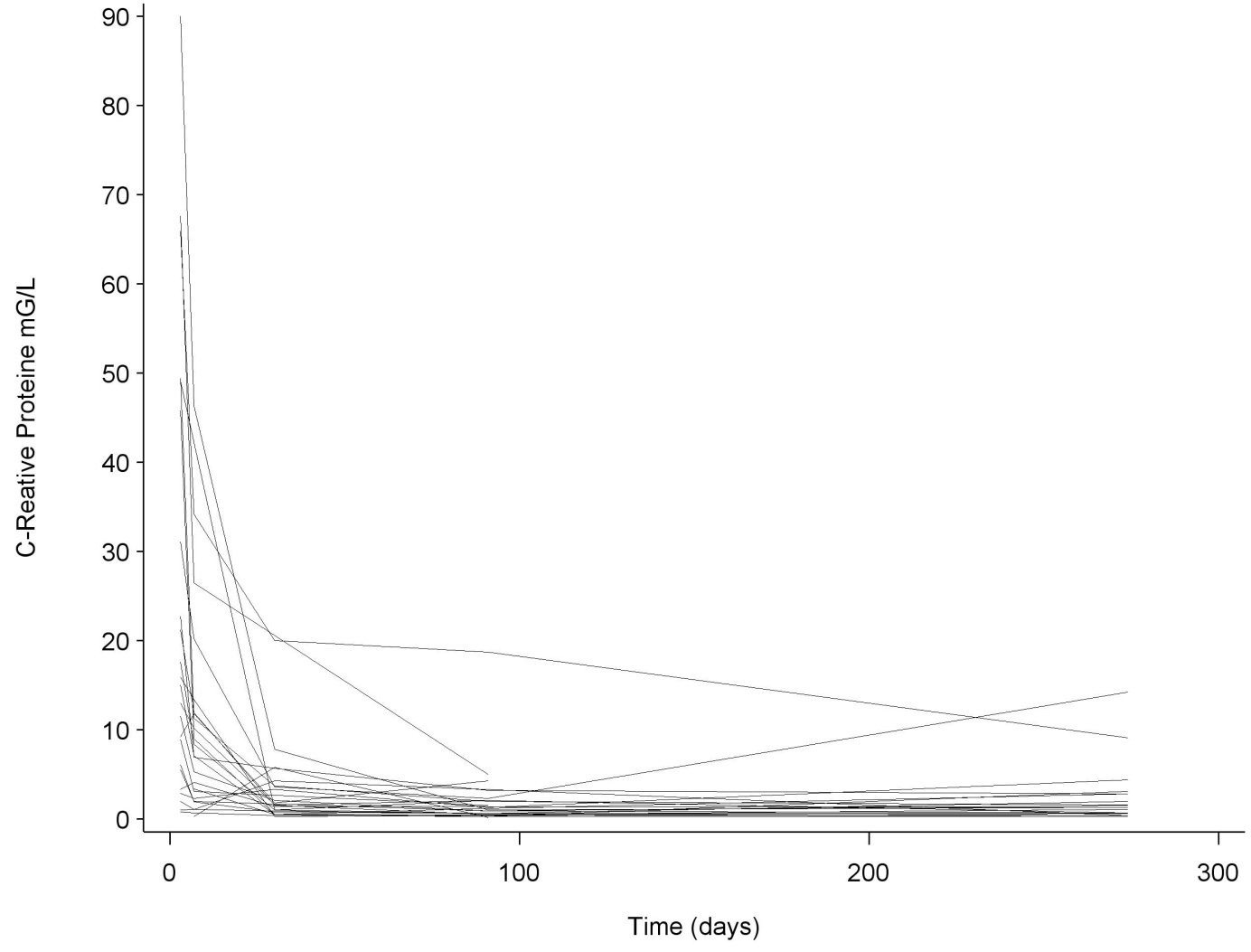


**Figure S2**: hsCRP evolution in patients with at least one MINI positive, during the 9-month follow-up period
